# Developing and streamlining clinical trial services to support pediatric drug development across 21 countries in Europe: insights from the conect4children (c4c) network

**DOI:** 10.3389/fmed.2025.1531276

**Published:** 2025-06-09

**Authors:** Sabah Attar, Francisca P. Figueiras, Carla Peacock, Hazel Wohlfahrt, Birgitta C. P. Hüsken, Susan Andrews, Pirkko Lepola, Margaret Patton, Regis Hankard, Eren Halil, Fernando Pontes Soares, Lionel K. Tan, Ricardo M. Fernandes, Mark A. Turner

**Affiliations:** ^1^Department of Women’s and Children’s Health, Liverpool Women’s NHS Foundation Trust, University of Liverpool, Liverpool, United Kingdom; ^2^conect4children Stichting, Utrecht, Netherlands; ^3^Association for Research and Development of Faculty of Medicine, Academic Consulting Research Organization, CETERA, Stand4kids, Lisbon, Portugal; ^4^Laboratory of Clinical Pharmacology and Therapeutics, Faculty of Medicine, University of Lisbon, Lisbon, Portugal; ^5^Johnson & Johnson, Breda, Netherlands; ^6^MPH, GSK, Durham, NC, United States; ^7^HUS Helsinki University Hospital and University of Helsinki, Helsinki, Finland; ^8^Novartis Pharmaceuticals Corporation, One Health Plaza, East Hanover, NJ, United States; ^9^PEDSTART, CHU Orléans, INSERM, F-45000, Orléans, France; ^10^Johnson & Johnson, High Wycombe, United Kingdom; ^11^IRIS-Servier, Gif-sur-Yvette, France; ^12^ViiV Healthcare, GSK HQ, London, United Kingdom

**Keywords:** pediatric drug development, clinical trials, trial services, performance management, feasibility, site identification, technology readiness levels, Service Readiness Levels

## Abstract

The efficiency, quality, and scalability of clinical trial support services are essential for the success of multinational clinical trials particularly trials that recruit babies, children, and young people. Through a public private partnership funded by the Innovative Medicines Initiative 2 between 2018 and 2025 involving 10 large originator pharmaceutical companies and 33 academic and third sector organizations, the conect4children (c4c) network has developed high-quality trial support services to promote consistent delivery in pediatric trials in over 220 sites across 21 countries, addressing gaps in communication, site identification, feasibility, and trial support. This paper explores the development and implementation of these services, using the Technology Readiness Levels (TRLs) and Service Readiness Levels (SRLs) frameworks to measure service progression and operational maturity. The initiative successfully streamlined targeted aspects of trial support. Over 6 years the multinational coordination of pediatric trials moved from SRL1 to SRL8 and services have been deployed in a sustainable non-profit organization. Challenges encountered include variability in site readiness for clinical trials and processes. Differences between companies in methodologies for collecting data about trial setup prevented clear understanding of the benefits of the c4c approach. Sustainability of long-term infrastructure beyond IMI2 funding will be managed by a new, independent, non-profit organization, conect4children Stichting based on scale up of services provided to industry and academia. The findings provide generalizable insights and lessons applicable to other research networks seeking to build or improve similar infrastructures.

## 1 Introduction

Pediatric clinical trials face unique challenges that differentiate them from adult trials, making their efficient and ethical conduct both essential and complex (1). Limited patient populations, heightened ethical considerations, and the need for specialized, experienced research sites create substantial barriers to trial execution (1). For children and their families, delays in bringing treatments to market can mean prolonged periods without effective therapies or adequate safety data for existing treatments. These delays in trial timelines also impact Europe’s standing in the global healthcare market (2–4). Some healthcare needs are immediate (e.g., treatment of COVID-19), others are long-term, e.g., post marketing surveillance. Addressing these issues requires streamlined, well-coordinated systems that can support pediatric clinical trials efficiently, effectively, and to high standards.

### 1.1 Role of c4c in enhancing pediatric clinical trials

The conect4children (c4c) network, established as a public-private partnership between 2018 and 2025 as a c4c project under the Innovative Medicines Initiative 2, IMI2. IMI2 and its successor the Innovative Health Initiative (IHI) were setup to promote collaboration between industry and academia to address public health needs, improve the lives of patients, and promote Europe’s health industries (5). c4c was commissioned as a Europe-wide clinical research initiative and addressed several activities including Expert Advice about pediatric drug development (6), work on pediatric data standards (7, 8), education (9), pharmacovigilance (10), and services to support clinical trials (this paper). The work described in this paper focused on addressing selected inefficiencies in pediatric clinical trials by creating a coordinated support network across Europe (11). A primary objective of IMI2 and IHI is to ensure the sustainability of projects like c4c, enabling them to continue the operational model beyond their initial project phases. Hence the developed network and service are to continue beyond 2025, as an independent not-for profit organization, registered in the Netherlands in April 2023, as conect4children Stichting (c4c-S). c4c-S receives fees for services from industry and through participation in grants. Stakeholders can also become Strategic Members who offer advice but have no role in governance; industry Strategic Members pay a membership fee.

One of the critical barriers in multinational clinical trials is the inconsistency in processes across different trial sites (i.e., hospitals and clinics) and countries, including fragmented communication, varying regulatory frameworks, and unaligned site readiness (12). Comprised of 10 large pharmaceutical companies and 37 non-industry partners—including academia, hospitals, third-sector organizations, and patient advocacy groups, c4c brings multi-stakeholder input into identifying concrete solutions for trial support services, strategically designed not only to address immediate challenges in pediatric research but also to create a self-sustaining infrastructure. At the time the project was established, IMI2 consortia could include countries in the EU and EEA, which in 2018 included the UK. Accordingly, the project did not address all possible problems in pediatric drug development, only those gaps that industry and academia could address through direct collaboration. IMI2 funding had two components: money allocated to the project by IMI that was managed by the project coordinator and Beneficiaries; in-kind contributions from the participating pharmaceutical companies. Industry partners provided in-kind contribution to the project by contributing expertise across all the work-packages.

The c4c network structure includes a supra-national Network Infrastructure Office (NIO), to oversee and direct activity, with a Single Point of Contact, (SPoC), within the NIO that is a central contact point for trial teams and internal members and National Hubs (NHs) as points of contact in each collaborative country. Each NH works with a national research network, connecting multiple trial sites at country level.

The c4c network services cover multiple stages of the clinical trial lifecycle—from protocol development, site finding, feasibility assessments, and support during trial conduct—providing the infrastructure needed to support both academic and industry-sponsored studies (13).

The c4c trial services were co-designed by both industry and academic partners within a structured governance model to support several stages of a clinical trial. These services provide guidance and coordination for trial teams but were designed to not involve any transfer of regulatory obligations to c4c. The viability of the network was assessed through Proof of Viability (PoV) trials, which tested the effectiveness of the services developed by the consortium. This included: three academic-led trials, which were funded by the consortium according to an independent, international peer-reviewed selection process and five industry-sponsored trials, which funded by the respective Sponsor. An additional four industry trials were adopted by the network during the c4c project.

By streamlining trial support and leveraging local knowledge and insight, c4c aims to ensure trials are conducted efficiently, maximizing the quality of study data and minimizing delays in trial setup and conduct. The collaboration between public and private partners, aims to ensure that both academic and industry perspectives contribute to the development and validation of trial services within the network. The ultimate benefits of this work will be successful trial completion and availability of new medicines for children. However, those benefits will take may years to materialize and a causal effect of the network’s work is difficult to assess with a small number of trials. Accordingly, this report focuses on the processes that need to be in place to underpin successful trial completion. The goal of this paper is to describe the development of c4c Trial Support Services using an explicit framework.

### 1.2 Rationale and relevance of technology readiness levels (TRLs) and Service Readiness Levels (SRLs)

TRLs were initially developed by the US Space Agency, NASA, to assess the maturity of technologies, tracking progression from basic research to deployment-ready innovations. TRL frameworks provide a structured method to evaluate the progress and maturity of the services developed across the trial lifecycle (14). The TRL framework ranges from early-stage research (TRL 1) to deployment-ready solutions (TRL 9). It was transformed into the International Organization for Standardization (ISO) standard 16290 (15). A simplified, list of technology readiness levels was adopted by the Horizon Europe programs to monitor research progress, including clinical trials and medical innovation projects (14, 16). TRLs have been adapted for the development of services (17) and utilized as Service Readiness Levels (SRLs) which emphasize service improvement and contribute to effective scaling and integration.

The paper aims to present the development and optimization of trial services within c4c. This includes work on trial services across c4c NHs during the project and the work done to deploy the trial services in the not-for-profit legal entity, c4c Stichting (c4c-S), that was established to implement services in a sustainable way. The objectives of this paper are to describe development, performance management, and lessons learned from ongoing trials.

## 2 Materials and methods

### 2.1 Context

The c4c Network including academic representatives from 21 countries representing 20 c4c NHs, with Iceland working with the Finnish NH, and 10 pharmaceutical industry partners co-developed selected trial support services across various stages. The project started in May 2018 and will close in April 2025. The activities described in this paper will be mapped to project months, with M0 denoting May 2018.

The development of the network will be described in another paper but in brief the NH model was selected in order to address national issues (ethics, National Competent Authorities, language) that are part of the communication issues that can hamper clinical trials. NH also provide local knowledge and relationships based on clinical ties rather than the transactional approach used by many commercial contributors to drug development. The academic leadership of the project made contact with potential NHs in 2016–2017 using a variety of routes including direct contact with networks already in existence, recommendations from members of the Pediatric Committee at the European Medicines Agency, recommendations from learned societies and specialist networks, and personal contacts. NHs were briefed about the developing network collectively and during face-to-face meetings in each country. A maturity matrix and quality standards were used to support network alignment. Peer-to-peer learning and action learning sets were also used (to be described in a separate paper).

The co-creation route for each of the developed services followed a clear procedure with industry, academic, country-level and site-level colleagues represented in task groups and incorporated a consultation (and subsequent revision) phase with all National Hubs and Industry partners.

#### 2.1.1 Identification of services needed

The work of the c4c network related to trial services began with a comprehensive needs assessment, identifying critical services required to support pediatric clinical trials across multiple countries and sites. Key gaps included the following main operational aspects:

• Communication and coordination: Fragmented communication between trial sponsors, National Hubs, and individual sites delayed trial initiation.
• Site identification and feasibility: Challenges in matching trial requirements with pediatric-specific site capabilities led to inconsistent site selection.
• Governance and quality assurance: Lack of standardized frameworks for monitoring and quality management of support provided at national or site level.

Stakeholders from academic institutions, industry sponsors, and trial coordinators provided input through structured meetings to define these service needs and set priorities. A first face to face meeting held in M10 of the project mapped needs and requirements. Further co-development in a larger group, consultation across wider set of partners, re-development to incorporate input from consultation was undertaken.

#### 2.1.2 Adapting the framework for c4c as SRLs

**FIGURE 1:**
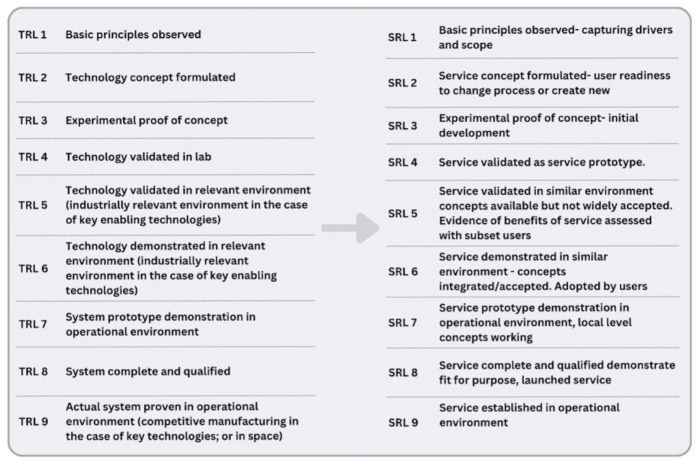
The following figure shows the agreed c4c descriptions for each SRL adapted for use and agreed by all task teams. It was noted that each description should be as broad as possible to ensure suitability for all workstreams to use.

The definitions of TRLs in were reviewed by the c4c task teams, which agreed to develop them as SRLs. The SRL framework shown in Figure 1 was used to evaluate each service from conceptualization through to full deployment. SRLs ensured that services such as site identification, trial feasibility and trial support were not only designed efficiently but were scalable and sustainable across countries.

#### 2.1.3 Pilot Testing using Proof-of-Viability trials

The c4c network implemented a total of 11 Proof-of-Viability (PoV) trials to test and refine services. Both academic and industry-led trials were used as pilots to gather real-world feedback on services. Early trials revealed areas requiring adjustment, such as data collection, clarifying roles and responsibilities, as well as the need for more comprehensive standards for National Hubs and sites.

The development of trial support services within c4c was carried out by comprehensively mapping out a service readiness pathway with a timeline and deliverables from multiple partners.

#### 2.1.4 Mapping development and pilot activities to the SRL framework

The SRL framework was applied to guide the service development process from SRL 1 (basic principles observed) to SRL 9 (fully operational services). Each service moved through three phases:

1. Conceptualization and initial development (SRL 1–3): Identifying service needs and piloting early prototypes.
2. Development and prototype stage (SRL 4–6): Refining services through iterative feedback cycles and performance evaluations.
3. Operational readiness and scaling (SRL 7–9): Full operational rollout across National Hubs with feedback loops to sustain and improve services.

SRL 1 to SRL 3: Conceptualization and initial development: During the early stages, the trial support services, such as site identification and feasibility assessments, began with a focus on defining key needs. Focused meetings with NHs, academic and industry partners were held between M4 and M6 to evaluate basic requirements. This stage maps to SRL 1–3, where early proof of concept takes place. Requirement of and initial set up of a governance framework and pilot service was undertaken during M3 and M13 (Figure 1). Initial pilots were academically sponsored trials in the following therapeutic areas; Paracetamol in Premature Babies, Kawasaki Disease Coronary Artery Aneurysm Prevention trial and the Prospective validation & clinical evaluation of a new posaconazole dosing regimen for children & adolescents with cystic fibrosis and Aspergillus, subsequently referred to as Academic PoV Trials 1, 2, and 3.

SRL 4 to SRL 6: development and prototype stage: The implementation of the services for the academic trials as initial proof of concept was undertaken across the network starting at M13 and with reviews at M20 and M22 based on experiences from academic trial teams, Network Infrastructure Office (NIO) and NHs. Improvements from were incorporated at M26.

From project month 21 (M21) onward industry sponsored trials were piloted, subsequently referred to as Industry PoV Trials 1 to 5. For the first Industry PoV trial, the NIO, National Hubs (NHs) implemented standardized processes with metrics and tools developed for site identification. This stage included use of tool and templates to validate the service in a controlled environment, including industry PoV trials, to test the robustness of c4c and NH systems across multiple countries.

SRL 7 to SRL 9: operational readiness and scaling

Ongoing support and quality assurance systems (metrics and NH and Site standards) were established across multinational sites, NHs transition to the final stages of SRL, from M38 onward. Further requests from industry sponsors who were also IHI project beneficiaries allowed “near-final” deployment of 4 additional industry studies, subsequently referred to as Industry Additional Trials 1, 2, 3 and 4. Legal frameworks and template agreements, and invoicing processes were developed for governance and service provision. These services became fully operational and ready for deployment across broader clinical trials, demonstrating scalability, as further discussed in the “3 Results” section.

Review of developed procedures and metrics would indicate if c4c and NHs services were consistently meeting their targets.

## 3 Results

### 3.1 Governance and quality framework

The governance and quality framework evolved from initial gap analyses into a comprehensive system that integrates monthly reviews of trial progress through service steps and continuous improvement mechanisms. This framework was designed to address operational oversight and ensure that all trial-related activities align with sponsor expectations. The framework progressed systematically through SRL 1–9, moving from conceptualization to full deployment across c4c. Annual performance reviews with NHs ensured ongoing compliance and continuous improvement, helping maintain service standards across the network. See Table 1.

**TABLE 1:** Evaluation of the governance framework against the Service Readiness Levels (SRL) framework.

Service Readiness Levels	Development work undertaken within SRL	Progress evidence
SRL 1–3: conceptualization and Pilot Testing	Need identified: Preliminary gap analyses revealed requirement of oversight of PoV trials across multiple hubs. Concept development: Governance structures were formulated and reviewed with stakeholders. Pilot projects: Tested governance models in hubs, with select trials, refining processes based on feedback.	Outcomes: Established and operationalized: 1. c4c Trial Committee (TrialComm) for governance of trials, c4c 2. Network Committee (NetComm) for governance of services, 3. Network Infrastructure Office (NIO), supra-national coordinator of service activity, established and functional. Early testing via Academic PoV studies indicated acceptability of framework across stakeholders and NHs.
SRL 4–6: validation and integration	Partial implementation: Initial governance tools (decision-making frameworks, escalation protocols) were used across some hubs, though inconsistently. Requirements for more consistent performance management framework noted. Requirement for revised governance frameworks and legal agreements [e.g., Confidentiality Disclosure Agreements, (CDAs)] for deployment of Industry PoV trials noted. Validation through feedback: Positive responses from early users confirmed value in the oversight processes, with performance reports showing improved monitoring. CDA’s and processes to deploy developed and timelines set. Integration: Templates and agreements were standardized across NHs, with the introduction of performance management framework for escalation.	Outcomes: Although validated by acceptance testing for use by all stakeholders, integration and uptake of performance management framework across NHs was variable. Standardization still needed across the entire network.
SRL 7–9: full deployment and continuous improvement	Operational rollout: Governance frameworks and tools established and with annual reviews and performance metrics ensuring oversight of all trial services and activities. Network-wide adoption: The final iteration of the governance framework for the sustainable new legal entity, c4c-S was officially launched after M60, with agreements that formed the framework to rollout out services seamlessly across NHs, guidelines, decision-making processes, and regular reviews in place. Sustained operations: Governance and legal frameworks were embedded as standard practice, ensuring a structure in place for contract renewals and risk management through continuous monitoring.	Outcome: Annual performance reviews demonstrated sustainability, scalability, and alignment with sponsor expectations. Operational templates for National Hub Cooperation Agreements, Master Service Agreements with Sponsors, Confidentiality Disclosure Agreements, Work Orders for use between c4c and Sponsors, Membership Agreements developed/adapted for use in the future sustainable non-profit legal entity.

### 3.2 Single Point of Contact, SPoC

The Single Point of Contact, SPoC, embedded within the supra-national Network Infrastructure Office, was set up to provide a central communication line within the organization to handle all user requests and coordinate all service activity across National Hubs. The maturity and scalability of the c4c SPoC service as it progressed through the SRL stages is captured in Table 2; Beginning with conceptualization (SRL 1–3), the service moved through pilots and validation through feedback (SRL 4–6), ultimately reaching full-scale deployment of processes with a custom built tool that was used for continuous operation (SRL 7–9). Each stage highlights the milestones achieved and the steps taken to ensure adoption, operational efficiency, and scalability.

**TABLE 2:** Evaluation of the Single Point of Contact (SPoC) service against the Service Readiness Levels (SRL) framework.

Service Readiness Levels	Development work undertaken within SRL	Progress evidence
SRL 1–3: conceptualization and pilot stage	Drivers identified: A streamlined approach to provide all trial-related services through a single contact point was identified. Communication gaps between National Hubs (NHs) and sponsors highlighted the need for a centralized contact service. Concept development: Initial task team meetings were held to design the SPoC workflows model, engaging stakeholders and gaining preliminary buy-in. Key mechanisms were identified- use of an email inbox and a dedicated software tool. A concept for the SPoC service was formulated during task team meetings (M2 to M6), with representatives from NHs and sponsor organizations participating. Pilot Testing: An initial prototype of the SPoC service was developed using an email inbox to manage communications. This prototype was implemented across all communications for the Academic PoV trials to test and refine workflows. During the initial phase, responses were tracked through spreadsheets. As part of continuous improvement, the decision was made to eventually implement a software tool with built-in service metrics and Key Performance Indicators (KPIs).	Outcomes: Limited adoption in early trials highlighted operational gaps that required further refinement.
SRL 4–6: validation and expansion	Local implementation: SPoC email prototypes were tested with Academic PoV trials. Implementation was across all NHs with consistent workflows. Performance tracking: Positive feedback confirmed faster communication and coordination improvements. Wider adoption: Multiple NHs integrated the service into their workflows, aligning it with SOPs and improving performance metrics. Key stakeholders, including trial sponsors and multiple hubs, accepted and integrated the SPoC model into their workflows.	Outcomes: Initial trials Academic Proof-of-Viability trials validated the feasibility of the SPoC model. SPoC demonstrated measurable benefits in trial management, but challenges remained in scaling the service across all communications with respect to data capture and proactive management of requests.
SRL 7–9: full deployment and continuous improvement	Operational rollout: The service became fully operational with a software tool deployed to support workflows and metrics data capture, across all NHs, trials subset increased to Industry PoV trials. Network-wide launch: NHs received formal training for the tool. Monitoring and improvement: Quarterly reviews and feedback loops ensured the service remained responsive and aligned with network goals.	Outcomes: SPoC reducing communication bottlenecks, with consistent workflows and enhancing engagement. Integral to trial operations across the network, achieving sustainability and continuous improvement.

### 3.3 Site identification and feasibility service

The site identification service was developed to streamline the selection of trial sites. A centralized database listing over 220 sites across 21 countries allowed NHs to quickly identify appropriate sites. The protocol specific feasibility used Trial-Specific Questionnaires (TSQs) with trial team input. These tools enabled efficient feasibility assessments, ensuring that trials were matched by NHs with capable pediatric sites and investigators that could meet the specific protocol requirements, leveraging NH local insights.

The service followed the SRL framework, progressing from local pilots (SRL 4) to full network-wide implementation (SRL 9) with timeline metrics in place. Ongoing internal reviews and real-time feedback loops ensured continuous improvements, helping NHs adjust processes to evolving trial needs.

**FIGURE 2:**
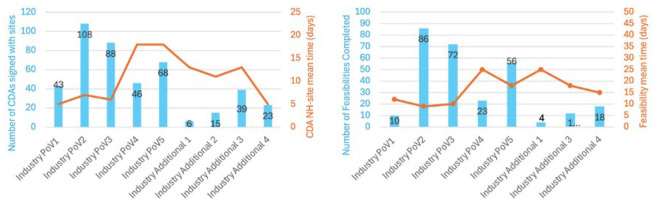
Charts showing number of sites that were set up with confidentiality disclosure agreements in place, and the number that completed protocol specific feasibility across c4c with the timelines for CDAs and completion of feasibilities at site level indicated. Industry Additional Study 2 did not progress to undertake feasibility or study opening.

The number of sites that were set up across 11 studies, with confidentiality disclosure agreements in place is demonstrated in Figure 2, including the number that completed protocol specific feasibility across c4c with the timelines for CDAs and completion of feasibilities at site level indicated. Industry Additional Study 2 did not progress to undertake feasibility or study opening.

### 3.4 Ongoing trial support service

The ongoing trial support service provided centralized coordination across countries and sites, ensuring consistent communication and rapid issue resolution. c4c NIO and NHs played a pivotal role in supporting recruitment strategies, site setup, and monitoring trial progress through monthly meetings.

The service evolved from early pilots (SRL 3–6) to full-scale deployment (SRL 9), becoming an essential component for seamless trial operations. NHs were instrumental in maintaining recruitment progress, resolving site-level challenges, and managing compliance across countries.

**FIGURE 3:**
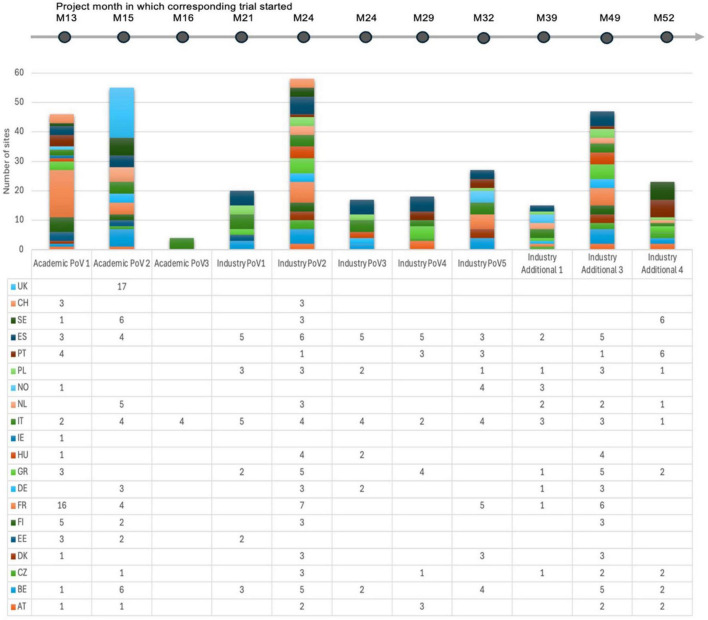
Schematic showing the number of sites and NHs that participated in each trial with an indication of the project month at which the respective trial started.

Figure 3 shows the number of sites and NHs that participated in each trial with an indication of the project month at which the respective trial started. The structured approach ensured effective site coordination, faster issue resolution, and continuous service enhancement. Metrics and performance management procedures ensured the network could undertake high-quality support of trial conduct, with escalation procedures and accountability, while aligning the framework with evolving operational and strategic goals.

## 4 Discussion

### 4.1 Impact on pediatric drug development

The conect4children (c4c) network model has the potential to advance pediatric drug development by supporting faster and more efficient clinical trials while maintaining high standards of quality and compliance. It addresses specific challenges in pediatric research, such as small patient populations and complex regulatory frameworks, by ensuring that trial sites are activated promptly and are well-prepared leveraging national and specialty specific insights and relationships. The network’s ability to rapidly deploy trial services is particularly critical for rare pediatric conditions, where streamlined processes can ensure timely delivery of new therapies to patients. The co-creation model of a public private partnership that the IHI grant enabled, allowed scoping a broad range of requirements at the outset across all relevant stakeholders. However, some misalignments posed limitations during the pilot rollout due to a lack of clarity in the service design phase, mainly due to industry partner representatives’ lack of insight into various other functions within their organizations, highlighting the importance of identifying role specific input into the scoping phase. The current work within the c4c network has not yet provided clear evidence of its impact on some outcomes, such as trial conduct timelines. This limited dataset of trials, within the constraints of the project duration, has meant that majority of them have been in the set-up phase. Hence, no definitive data has shown that these services have significantly reduced the time required to set up, conduct, or complete trials. Furthermore, the framework has not yet demonstrated that it speeds up or facilitates the process of obtaining regulatory approval or licensing for new drugs or therapies by the regulatory authorities. Nevertheless, the platform has been established in a sustainable form and is ready for scale-up.

The needs and opportunities for pediatric drug development are hampered by variation in site readiness and initiation. Coordination, such as the work of c4c, is part of the solution but site optimization is also required. This requires drivers and incentives for sites that are often overwhelmed with clinical care.

As shown elsewhere, the data collected about clinical trials varies substantially between clinical research networks (18). This divergence appears to relate to the drivers for performance management and purposes of data collection. We suspect that the divergence in industry datasets reflects similar influences; harmonizing data collection about trial processes would require significant resources. The lack of process metrics increases the importance of outcome metrics, in this case the timely completion of trials. As noted, the sample of trials supported by c4c is too small to draw firm conclusions. Focusing on individual metrics may give an incomplete picture. The work of c4c could also benefit trials by facilitating work and thus reducing costs.

The network could influence regulatory procedures but this requires Sponsors to pass information from the network to the regulators, e.g., the Pediatric Committee at the European Medicines Agency. Before a Pediatric Investigation Plan is finalized, input from sites and NH could provide understanding of patient flow and standards of care across Europe that could contribute to the design of feasible plans and protocols (19). Similarly, if a protocol is in difficulty, input from sites and NH could help identify studies that are impossible due to an absence of patients.

The impact of c4c arises from timely, high-quality access to a broader range of pediatric sites than companies generally have access to. This paper does not describe the business model of the non-profit but work during the project has identified fees for service to industry that are affordable to the project and sustainable for the non-profit.

c4c-S does not deliver services that require delegation under Good Clinical Practice for clinical research: there are no Transfers of Regulatory Obligations during c4c-S services. c4c-S avoids duplicating the work of specialized trials networks and its services for each engagement are adapted to avoid duplication with work done by industry or academic Sponsors. Companies cannot employ experts in all pediatric conditions: c4c provides consolidated access to the full breadth of expertise.

### 4.2 Operational readiness and performance management

Challenges related to site responsiveness and recruitment bottlenecks were significant, especially as the academic PoV trials were setting up and opening to recruitments during the time COVID-19 pandemic; this had a potent impact to public community, hospitals’ human resources and normal trial conduct worldwide. The c4c network addressed these issues by implementing business continuity planning during the COVID-19 pandemic, monthly progress meetings and issue escalation procedures to manage delays efficiently. One challenge with the performance management framework was that the intended real-time dashboards and tracking tools were not ready until the final year of the project. As a result, much of the performance reporting had to rely on manual data collection via monthly reports and spreadsheets completed by sponsors and trial teams.

While the academic PoV trials demonstrated good adherence to these reporting requirements, industry trials were less consistent in providing complete datasets due to the companies having different existing trial data collection systems and methods for data capture and monitoring. This created some inconsistencies in the representation of trial metrics at both the international (NIO) and national (NH) levels, which impacted real time proactive problem solving during earlier phases.

The introduction of real-time dashboards in the final year has the potential to enhance monitoring capabilities, provide greater visibility into site performance, recruitment progress, and issue management (Table 3).

**TABLE 3:** Evaluation of the ongoing trial support service against the Service Readiness Levels (SRL) framework.

Service Readiness Levels	Development work undertaken within SRL	Progress evidence
SRL 1–3: conceptualization and Pilot Testing	Need identified: Identified gaps in site coordination, issue tracking, and monitoring, leading to the conceptualization of a communication cycle. Pilot Testing: The service was piloted with limited tools (spreadsheets and reports) to test communication and issue escalation processes. Need identified: Gaps in consistent quality across hubs led to the development of monthly meetings with NHs working on trials. Pilot Testing: Pilots introduced key elements like SOPs and performance metrics (e.g., timelines), but initial trials identified challenges in collecting and reporting metrics.	Outcomes: Pilots showed early success but highlighted the need for more robust tracking systems. Early pilots validated the manual workflows for information gathering but indicated the need for refined tools and processes.
SRL 4–6: validation and integration	Partial adoption: NHs implemented the service with mixed results, showing improvements in trial monitoring but inconsistencies in delivery. Select NHs integrated the framework, showing measurable improvements such as faster site activation and more effective audits. Feedback integration: NH and sponsor feedback helped to refine processes and improved consistency across hubs, though full-scale adoption was still ongoing.	Outcomes: Performance reviews confirmed initial benefits, though full-scale adoption across the network was still in progress. Metrics for monthly tracking of recruitment and site activities demonstrated early success, but additional adoption and standardization across all hubs were needed.
SRL 7–9: full deployment and continuous improvement	Operational rollout: The service was launched across all NHs with a comprehensive quality framework and communication cycle with three sets of monthly meetings between NIO and Sponsor, NIO and NHs and each NH and its sites, covering quality management and improvement, functioning as a standard offering, with regular feedback loops resolving site-level challenges. SOPs, templates, and performance dashboards were implemented, with quarterly reviews ensuring compliance and improvement. Network-wide adoption: All hubs and sites were trained in the service, with near real-time dashboards and quarterly performance reviews ensuring continuous monitoring and improvement.	Outcomes: Reviews demonstrated the sustainability and impact of the service, with consistent engagement from sponsors. Performance Management and Feedback loops: Established communication cycle and regular reviews between NHs, sponsors, and trial teams ensured responsiveness to evolving trial needs.

By consistently meeting the 20-day site identification targets (Table 4) and standardizing contracting processes, c4c demonstrated its ability to respond flexibly to evolving demands, highlighting the maturity of c4c services and aligning with Service Readiness Levels (SRL) 7–9, where operations are fully validated and ready to support all academic and commercial trials.

**TABLE 4:** Evaluation of the site identification and feasibility service against the Service Readiness Levels (SRL) framework.

Service Readiness Levels	Development work undertaken within SRL	Progress evidence
SRL 1–3: conceptualization and Pilot Testing	Need identified: Challenges in site selection led to the creation of a structured service for site identification and rollout of Trial-Specific Questionnaires (TSQs) for protocol specific feasibility. Pilot Testing: A prototype service was trailed with limited hubs participating in feasibility for Academic Proof-of-Viability (PoV) studies, which highlighted areas for improvement (e.g., data entry).	Outcomes: Early trials showed feasibility but also revealed inconsistencies that required refinement.
SRL 4–6: validation and integration	Early implementation: The creation of a centralized database of all sites facilitated site identification for Industry PoV trials. The site identification and protocol feasibility services were adopted across all NHs integrating it into workflows. Validation: Positive feedback confirmed the service’s benefits (faster site selection timelines) but highlighted the need for broader adoption. Integration: Sponsors actively began using the service, with c4c and NHs tracking performance metrics (e.g., site identification within 20 days).	Outcomes: Improved communication and alignment on site capabilities, though fine-tuning was required for full-scale adoption. Site ID database functional. An innovative Confidential Disclosure Agreements, CDA cascade process requiring only one agreement from sponsor to SPoC, further expedited trial startup, reflected an increasing maturity in operational tools from M21 onward. This fits within SRL 4–6, where the service is validated in a controlled environment.
SRL 7–9: full deployment and continuous improvement	Operational rollout: The service became fully operational across all NHs, resolving local challenges and establishing best practices. Network-wide adoption: Standard Operating Procedures (SOPs) ensured consistency, and regular audits confirmed the service was fit for purpose. Sustainability: Continuous monitoring and feedback loops were implemented to sustain service quality and responsiveness.	Outcomes: The service became an essential component of trial delivery, with performance reports confirming its scalability and effectiveness. Sites were consistently identified and selected within 20 days, meeting efficiency goals.

### 4.3 Governance and communication models

The adoption of the Responsible, Accountable, Supportive, Consulted, Informed (RASCI) model for defining and allocating activities played a pivotal role in improving collaboration across stakeholders by clearly defining roles and responsibilities for sponsors, CROs, NHs, and trial teams. The need for this originated during the initial scoping calls with one of the Industry PoV trials as it became apparent that there was overlap at country level in activities and functions between the NH staff and the local representatives of Sponsors. The decision was taken to use the RASCI convention to develop the matrix rather than the more conventional RACI, as there are multiple activities that c4c undertakes which are “Supportive.” It was designed with NH and industry colleagues to capture the complexities of defining roles for multiple organizations in trial processes. The pilot was undertaken across NHs and sites for a subset of Industry trials, one of which included a Contract Research Organization. The findings indicated that the RASCI minimized operational delays and ensured smoother communication throughout the trial lifecycle.

The NIO also conducted training workshops with NHs to prepare sites for trial responsibilities, ensuring alignment of expectations. These were also based on real time experiences of an Industry PoV trial. Real-time communication between NHs and sponsors facilitated faster issue resolution, as demonstrated by adjustments made during the Industry PoV trials 1, 2 and 3, which highlighted the need for enhanced communication cycles integrated to maintain trial timelines. There was positive feedback on the subsequent implementation of the RACSI matrix to clarify roles and responsibilities, from global sponsor teams as well as NHs and country specific sponsor representatives.

### 4.4 Lessons for future pediatric clinical research

The lessons learned from current trials will inform the design and execution of future pediatric research, especially in rare conditions through the integration of c4c’s work with other networks. Currently alignment work with European Reference Networks (ERNs), which are also cross-border networks focused on rare or complex diseases could allow for improved collaboration and potentially streamline processes across these networks.

A close alignment with European Network of Pediatric Research at the European Medicines Agency (EnprEMA) throughout the life of the project has ensured participation in efficient inter-network and stakeholder collaboration. As a recently registered member of EnprEMA and its Coordination Group (since 2025), conect4children Stichting is set to continue this collaborative partnership. c4c works through EnprEMA, and independently, to respond to consultations about regulatory guidelines.

The ability to overcome recruitment challenges, streamline site setup, and maintain high operational standards, whilst ensuring alignment across key stakeholder groups and initiatives, provides a roadmap for other research networks.

These experiences emphasize the importance of flexibility and iterative refinement in trial management, particularly in multinational settings. Moving forward, the focus will remain on ensuring that these services continue to support both academic and commercial trials effectively in the new legal entity deployment, conect4children Stichting, whilst undertaking more work to gather evidence on the impact of these services on timelines and licensing.

### 4.5 Using the Service Readiness Levels (SRLs) framework for developing services for clinical trial support

The implementation of the Service Readiness Levels (SRLs) framework within the c4c network offered several important insights into how clinical trial services can mature systematically. While the SRLs provided a valuable structure for tracking progress from conceptualization to full-scale deployment, several key lessons emerged along with some conceptual and methodological constraints.

Clarity and planning at early SRL stages: A critical challenge in applying the SRL framework was the lack of clarity during the early planning stages (SRL 1–3). The need to define clear service requirements early on became evident during the pilot phases, where misalignments in initial design surfaced. For future projects, a more robust needs assessment and stakeholder alignment at SRL 1 would help to minimize revisions later in the process.

Balancing pilot flexibility with timely rollouts: The pilot phases (SRL 4–6) demonstrated the importance of flexibility and real-world testing. However, delays in deploying SPoC tool until 4th year of project (2021) and integrated dashboards until the final year (2024) of the project revealed the challenge of managing dependencies on technology readiness.

Adaptability through feedback loops: The SRL framework emphasizes continuous learning and feedback mechanisms. In the c4c model, feedback loops were embedded at multiple levels—within NIO, NHs, sites and sponsor communications. These loops allowed the network to adapt communication strategies and issue management processes dynamically, contributing to better performance over time.

Integrating governance and quality monitoring: A significant takeaway from the c4c experience is that governance and quality assurance frameworks must evolve alongside operational services. Basic versions of these need to be available prior to pilot services being deployed. The delay in real-time dashboards highlighted how performance management systems need to be integrated early. However, the adoption of manual interim solutions, such as monthly reports, ensured continuous monitoring despite delays.

Addressing these constraints required iterative refinement, adaptive use of the SRL model, and frequent recalibrations of the framework to accommodate service-specific needs and the collaborative requirements inherent in a large-scale, multinational research network.

Recommendations to developing clinical research networks:

• Introduce workshops and stakeholder consultations with specific requirements identified at SRL 1–2 to ensure all service components are well-defined.
• Develop detailed conceptual roadmaps early, including clear milestones aligned with each SRL stage.
• Technology development timelines should align with service rollouts to avoid reliance on interim manual processes.
• Use parallel pilot and development tracks where feasible to test key service components sooner.
• SRL stages should incorporate structured feedback reviews at each transition point to ensure ongoing improvement. Iterative improvements were critical in the success of c4c service development.
• Performance tracking tools must be aligned with governance frameworks from the outset to avoid gaps in data monitoring and decision-making.
• Interim solutions (like manual reports) can maintain oversight but must be clearly defined and managed to avoid overburdening stakeholders.

Generalizability for other clinical research networks: Applying the SRL framework to the c4c network has demonstrated its value as a generalizable model for other clinical research networks. It offers a structured methodology for tracking service development across diverse trials, ensuring that projects are scalable, operational, and sustainable.

However, networks considering SRL implementation should be aware of potential delays if technologies or services are not fully aligned with SRL timelines. Flexibility is essential, particularly when scaling services across multinational environments with varying regulatory and operational conditions.

## 5 Conclusion

Applying Service Readiness Levels (SRLs) to clinical trial services development offered a structured, measurable approach to evaluating how far services have come and what is required for full operational readiness. c4c adapted and adopted this framework to effectively scale operations, streamline site identification, feasibility and improve trial delivery across different countries. The introduction of standardized trial support services has enhanced operational efficiency, particularly in the setup phase, but key challenges remain in demonstrating the long-term impact on trial timelines and regulatory approvals.

While the SRL framework proved to be a robust methodology for tracking and guiding service development within the c4c network, early-stage misalignments in service design and industry engagement highlighted the need for clearer role delineation and early stakeholder alignment. The lack of real-time performance monitoring tools in the early phases of implementation led to reliance on manual reporting, delaying the ability to assess trial progress dynamically. By embedding feedback loops, scalable governance models, and continuous improvement mechanisms, c4c was able to overcome challenges and refine operations in real-time.

The network was developed by identifying shared goals, enlisting organizations with an appetite for change, quality, and efficiency, and by promoting a culture of performance managed collaboration.

Moving forward, research networks can draw from these lessons to design and deploy efficient, flexible, and sustainable trial services, ensuring readiness for both academic and industry-led clinical research. c4c-S provides an example for sustainable networks by systematically building services that meet pre-specified needs.

This blueprint can help ensure trial services are both efficient and adaptable to changing trial landscapes, setting the foundation for successful global research networks.

## Data Availability

The original contributions presented in this study are included in this article/supplementary material, further inquiries can be directed to the corresponding author.
